# ﻿Morphological characteristics and phylogenetic analyses revealed four new wood inhabiting fungi (Agaricomycetes, Basidiomycota) in Xizang Autonomous Region, China

**DOI:** 10.3897/mycokeys.106.125831

**Published:** 2024-06-25

**Authors:** Hong-Min Zhou, Xun-Chi Zhang, Jie-Ting Li, Fang Wu, Chang-Lin Zhao

**Affiliations:** 1 The Key Laboratory of Forest Resources Conservation and Utilization in the Southwest Mountains of China Ministry of Education, Key Laboratory of National Forestry and Grassland Administration on Biodiversity Conservation in Southwest China, Yunnan Provincial Key Laboratory for Conservation and Utilization of In-forest Resource, Southwest Forestry University, Kunming 650224, China; 2 College of Forestry, Southwest Forestry University, Kunming 650224, China; 3 Key Laboratory of Forest Ecology in Tibet Plateau, Ministry of Education, Institute of Tibet Plateau Ecology, Tibet Agricultural & Animal Husbandry University, Nyingchi, Tibet 860000, China; 4 Institute of Microbiology, School of Ecology and Nature Conservation, Beijing Forestry University, Beijing 100083, China

**Keywords:** Molecular systematic, phylogenetic analysis, taxonomy, wood-decaying fungi

## Abstract

Four new fungi from Xizang in southwest China, *Caloceraramaria*, *Ceraceomycesrhizomorphus*, *Leptosporomyceslinzhiensis*, and *Ramariaxizangensis* are described and illustrated based on the morphological and molecular evidence. *Caloceraramaria* is characterized by the ramal and bright orange basidiomata, a monomitic hyphal system with simple septa generative hyphae, usually 4-septate basidiospores; *Ceraceomycesrhizomorphus* is characterized by the cream to yellowish basidiomata with rhizomorphs, cylindrical basidiospores; *Leptosporomyceslinzhiensis* is characterized by white with pink basidiomata, cylindrical to oblong ellipsoid basidiospores; *Ramariaxizangensis* is characterized by flesh pink basidiomata, branched dichotomously in 4–5 ranks, a monomitic hyphal system with clamped generative hyphae, ellipsoid to cylindrical and densely warted basidiospores.

## ﻿Introduction

The fruiting bodies of Basidiomycota exhibit complex forms, such as gilled, poroid, toothed, coralloid basidiomata. Numerous taxonomists have endeavored to construct a stable classification system based on these characters (Gäumann 1953). Recently, the analysis of DNA sequences has emerged as a common method for deducing fungal phylogenies and enhancing higher classification frameworks through the integration of genetic traits (Cui et al. 2019; Wijayawardene et al. 2020; Liu et al. 2023).

The abundance of biodiversity in *Abies* forests can be attributed to the plentiful presence of humus and mycorrhizal fungi, which foster an optimal environment for the proliferation of the macrofungal species. Information regarding the fungal diversity in *Abies* communities is scattered over a range of publications (Ryvarden and Gilbertson 1993; Dai 2022). *Ceraceomyces* Jülich, a small genus characterized by yellow rhizomorphic basidiomata, was established by Jülich based on the taxon *C.tessulatus* (Cooke) Jülich (Jülich 1972). This genus, originally from North America, features annual, resupinate, pellicular basidiomata with a smooth or merulioid hymenial surface, a monomitic hyphal system, narrowly clavate basidia, and subglobose, narrowly ovate, ellipsoid to cylindrical basidiospores (Chikowski et al. 2016). Phylogenetic studies revealed that *Ceraceomyces* was polyphyletic, comprising three distinct groups. The section of *Corticiumtessulatum* Cooke belonged to Polyporales, and *Ceraceomycesserpens* (Tode) Ginns and *C.eludens* K.H. Larss. were part of phlebioid clade (Larsson et al. 2004). A recent study indicated that the type species, *Corticiumtessulatum* is classified under the order Amylocorticiales (Binder et al. 2010; Chikowski et al. 2016), as well as species, *C.yunnanensis* Qi Yuan & C.L. Zhao and *C.borealis* (Romell) J. Erikss. & Ryvarden (Yuan et al. 2023). Currently, eleven species are recognized in the genus *Ceraceomyces*, including *C.cystidiatus* (J. Erikss. & Hjortstam) Hjortstam, *C.eludens*, *C.microsporus* K.H. Larss. and *C.sublaevis* (Bres.) Jülich were accepted in the genus. A genus, *Crystallicutis* El-Gharabawy, Leal-Dutra & G.W. Griff. was derived from *Ceraceomyces* based on the crystals in the hymenium and subiculum of the basidiomata, which includes the brown-rot species *C.serpens* (El-Gharabawy et al. 2021). Both species *C.sulphurinus* and *C.violascens* (Fr.) Jscens were recorded in *Ceraceomyces*, are considered congeneric with *Rhizochaete* Gresl., Nakasone & Rajchenb. due to the characteristics like the rhizomorphic margin and the purple reaction in KOH.

*Calocera* (Fr.) Fr. is known for its distinctive characteristics, stipitate, fasciculate or scattered, gelatinous basidiomata, dendroid or staghorn-like, subclavate to clavate basidia and probasidia, as well as cylindrical to reniform, septate or non-septate basidiospores (Fisher 1931; Lowy 1971; Peng et al. 1992; Wu et al. 2011; Shirouzu et al. 2017). Recent phylogenetic analyses of the class Dacrymycetes demonstrated that *Calocera* was polyphyletic and species in the genus are scattered throughout the family Dacrymycetaceae together with most of the species of *Dacrymyces*Nees (1817: 89) as well as a few species from other genera such as *Dacryopinax* G.W. Martin (1948: 116) and *Femsjonia* Fr. (Shirouzu et al. 2007; Zamora and Ekman 2020).

The genus *Leptosporomyces* Jülich is characterized by the resupinate basidiomata, white yellow and smooth hymenial surface, a monomitic hyphal system with clamped connections, and thin-wall, smooth, acyanophilous basidiospores. Recent research has indicated that *Leptosporomyces* was polyphyletic, with two taxa, *L.galzinii* (Bourdot) Jülich and *L.raunkiaeri* (M.P. Christ.) Jülich, grouped in the order Atheliales, while *L.septentrionalis* (J. Erikss.) Krieglst. was placed in the order Amylocorticiales (Larsson 2007; Hodkinson et al. 2014; Sulistyo et al. 2021). The generic delimitation of *Fibulomyces* Jülich and *Leptosporomyces* remains controversial, with both being indistinguishable in phylogenetic and morphological analyses, leading to the former being considered as a synonym of the latter (Bernicchia and Gorjón 2010).

*Ramaria* Fr. ex Bonord. is a widely distributed non-gilled Basidiomycete genus (Marr and Stuntz 1973; Petersen 1981; Humpert et al. 2001). The genus is recognized by branched basidiomata, mono- to dimitic hyphal systems with clamped or simple-septate generative hyphae, and smooth to echinulate, verrucose-reticulate or striate ornamentation basidiospores (Corner 1950; Marr and Stuntz 1973; Petersen 1981; Humpert et al. 2001). The genus has been classified into four subgenera, namely R.subg.Ramaria, R.subg.Laeticolora Marr & D.E. Stuntz, R.subg.Lentoramaria Corner, and R.subg.Echinoramaria Corner (Marr and Stuntz 1973; Humpert et al. 2001; Exeter et al. 2006; Knudson 2012; Hanif et al. 2019). Initially, *Ramaria* was treated as a subgenus within *Clavaria* (Coker 1923; Doty 1944) until Corner (1970) elevated it to genus rank. Studies based on the morphological and molecular data agree on the paraphyletic state of *Ramaria* (Humpert et al. 2001; Hosaka et al. 2006; Giachini et al. 2010).

In the present paper, species from four genera are collected from Xizang under forest of *Abies*, and the phylogenetic relationships of four taxa are still unclear. Thus, to explore the diversity and taxonomic status with different characters for those taxa will be significant for macrofungi in Xizang, and the taxonomy and phylogeny analysis show that they are new to science.

## ﻿Material and method

The specimens were collected from Xizang which were deposited in the herbarium of the Southwest Forestry University (SWFC), Kunming, Yunnan Province, China. Samples were photographed when fresh in the field, and their habitats were recorded. Microscopic structures were discussed by Zhao et al. (2023). Special color terms were set by Anonymous (1969) and Petersen (1996). A Nikon Digital Sight DS-L3 or Leica ICC50 HD camera (magnification ×1,000) was used to exam hand-cut sections of basidiomata, which were first treated with 5% KOH for a few minutes and then with 1% phloxine B (C_20_H_4_Br_4_Cl_2_K_2_O_5_). At least 30 basidiospores of each species were examined. The values were expressed as a mean with 5% of the measurements excluded from each end of the range, given in parentheses. Stalks were excluded for basidia measurement, and the hilar appendages were excluded for basidiospore measurement.

### ﻿DNA extraction, amplification, and sequencing

The CTAB rapid plant genome extraction kit-DN14 (Aidlab Biotechnologies Co., Ltd., Beijing) was used to obtain DNA from dried specimens and PCR was performed according to the manufacturer’s instructions with some modifications (Yang et al. 2023). ITS were amplified using the primer pairs ITS5/ITS4 (White et al. 1990). The PCR procedure for ITS was as follows: initial denaturation at 95 °C for 3 min, followed by 35 cycles at 94 °C for 40 s, 54 °C for 45 s, and 72 °C for 1 min; and a final extension at 72 °C for 10 min. The PCR procedure for LSU was as follows: initial denaturation at 94 °C for 1 min, followed by 35 cycles at 94 °C for 30 s, 50 °C for 1 min, and 72 °C for 1.5 min; and a final extension at 72 °C for 10 min. All newly generated sequences were submitted to GenBank and are listed in Table 1.

**Table 1.** Taxa information and the sequences used in this study. *Newly generated sequences for this study.

Species	Locality	Voucher	ITS	LSU
*Amyloatheliacrassiuscula*	Sweden	GB/K169-796	DQ144610	–
*Amylocorticiumcebennense*	USA	HHB-2808	GU187505	GU187561
*Amylocorticiumsubincarnatum*	Sweden	GB/AS-95	AY463377	AY586628
*Amylocorticiumsubsulphureum*	USA	HHB-13817	GU187506	GU187562
*Anomoporiabombycina*	USA	CFMR L-6240	GU187508	GU187611
*Anomoporiavesiculosa*	China	Dai 22795	ON413718	ON413720
*Atheliaabscondita*	USA	Goyette 633	OP877120	OP902328
*Athelopsissubinconspicua*	Sweden	GB0058732	LR694197	LR694174
*Bondarzewiaoccidentalis*	Canada	AFTOL-ID 452	DQ200923	DQ234539
*Byssocorticiumcaeruleum*	Canada	RS 09400 (H)	NR_121454	–
*Calocerabambusicola*	China	Wu 9910-12	FJ195751	–
*Caloceracornea*	Sweden	UPS F 940775	MN595627	MN595627
*Caloceracornea*	Unknown	AFTOL ID 438	AY789083	AY701526
*Caloceracornea*	Sweden	UPS F 940774	MN595626	MN595626
*Caloceracornea*	Canada	CBS 124.84	AB712437	AB472738
*Caloceraguepinioides*	New Zealand	PDD 107969	LC131411	LC131370
*Caloceraguepinioides*	New Zealand	PDD 107981	LC131412	LC131371
*Caloceraguepinioides*	New Zealand	PDD 105005	LC131407	LC131366
*Caloceraguepinioides*	New Zealand	PDD 107874	LC131409	LC131368
*Caloceraguepinioides*	New Zealand	PDD 105033	LC131408	LC131367
*Caloceraguepinioides*	New Zealand	PDD 107929	LC131410	LC131369
*Caloceralutea*	New Zealand	PDD 107841	LC131413	LC131372
*Caloceralutea*	New Zealand	PDD 107842	LC131414	LC131373
*Calocerapalmata*	New Zealand	PDD 107830	LC131415	LC131374
*Calocerapalmata*	New Zealand	PDD 107925	LC131416	LC131375
*Calocerapalmata*	Japan	CBS 127.51	MH856777	MH868295
*Caloceraramaria*	China	CLZhao 31166	**PP399147**	**PP862915**
*Calocerasinensis*	China	MHHNU30743	MK167408	–
*Calocerasinensis*	China	Wu 0505-3	FJ195753	–
*Calocerasinensis*	China	Wu 0703-6	FJ195754	–
*Calocerasinensis*	China	JCH 070726	FJ195755	–
*Caloceratibetica*	China	Dai 20171	MW549777	MW750403
*Caloceratibetica*	China	Dai 20178	MW549778	MW750404
*Caloceraviscosa* *s.lat.*	Sweden	UPS F-940773	MN595628	MN595628
*Caloceraviscosa* *s.lat.*	Germany	FTOL ID1679	DQ520102	DQ520102
*Ceraceomycesamericanus*	USA	FP-102188	KP135409	KP135277
*Ceraceomycesatlanticus*	Brazil	URM 85888	NR_153926	NG_060427
*Ceraceomycesatlanticus*	China	M67	OR766067	KX685874
*Ceraceomycesborealis*	Sweden	KHL 8432	EU118610	–
*Ceraceomyceseludens*	Sweden	JS 27108	AF090879	–
*Ceraceomyceseludens*	Sweden	JS 22780	AF090877	–
*Ceraceomyceseludens*	United Kingdom	KM 194563	OR907143	–
*Ceraceomycesmicrosporus*	USA	UC 2023077	KP814418	–
*Ceraceomycesmicrosporus*	Sweden	JS 27153	AF090873	–
*Ceraceomycesrhizomorphus*	China	CLZhao 31154	**PP399151**	–
*Ceraceomycesrhizomorphus*	China	CLZhao 31161	**PP399148**	–
*Ceraceomycesrhizomorphus*	China	CLZhao 31188	**PP399149**	**PP862917**
*Ceraceomycesrhizomorphus*	China	CLZhao 31197	**PP399150**	**PP862916**
*Ceraceomycessublaevis*	USA	FP-101245-Sp	KP135029	GU187607
*Ceraceomycestessulatus*	USA	MPN 152885038	OR680647	–
*Ceraceomycestessulatus*	Sweden	KHL 16429	KU518951	–
*Ceraceomycesyunnanensis*	China	CLZhao 18992	OQ132519	OQ147003
*Clavariadelphusamplus*	China	HMAS 250466	MK705858	MK704448
*Coniophoramarmorata*	Belgium	MUCL: 31667	GU187515	GU187571
*Dacrymyceslongistipitatus*	New Zealand	PDD 107996	LC131425	LC131386
*Dacrymycespachysporus*	New Zealand	PDD 105004	LC131429	LC131392
*Dacrymycesparastenosporus*	New Zealand	PDD104960	LC131431	LC131394
*Dacrymycesstillatus*	Sweden	UPS F-939814	MN595676	MN595676
*Dacrymycessubalpinus*	Japan	TUFC12834	AB712465	AB299060
*Dacryonaemamacnabbii*	Sweden	UPS F-940949	MN595650	MN595650
*Dacryonaemamacnabbii*	Sweden	UPS F-940951	MN595651	MN595651
*Dacryonaemamacrosporum*	Norway	O 160045	MN595659	MN595659
*Dacryonaemamacrosporum*	Finland	UPS F-940998	MN595660	MN595660
*Dacryonaemarufum*	Sweden	UPS F-941003	MN595645	MN595645
*Dacryonaemarufum*	Sweden	UPS F-941005	MN595646	MN595646
*Dendrdacrysbrasiliense*	Brazil	INPA:241458	AB744230	AB723514
*Dendrdacrysdendrocalami*	Japan	TUFC 13914	AB712453	AB712428
*Fibulomycesmutabilis*	Germany	HG-B 5753 (GB)	GQ162817	–
*Ganodermaresinaceum*	Unknown	C45	KX371982	KX372027
*Gautieriaparksiana*	USA	SNF 236 USA	AF377059	–
*Gloeocantharellusneoechinosporus*	China	GDGM 75321	MK358820	MK358815
*Go.ludovicianus*	USA	TFB 14476	KJ655570	KJ655580
*Gomphusclavatus*	Spain	MA-Fungi 48085	AJ292292	–
*Hypochniciellumsubillaqueatum*	Sweden	KHL 8493	AY463431	AY586679
*Hypochniciellumsubillaqueatum*	UK	KM165142	MZ159402	–
*Kaviniahimantia*	USA	CFMR: DLL2011-079	KJ140598	–
*Kaviniaalboviridis*	USA	CFMR: DLL2011-131	KJ140634	–
*Lentariamicheneri*	USA	RRD6 (TENN)	MF773634	–
*Lactarius* sp.	New Zealand	PDD:113066	MW683864	MW683691
*Lentariabyssiseda*	USA	TENN 61159	FJ596788	–
*Leptosporomycesfuscostratus*	USA	UC 2022884	KP814350	–
*Leptosporomycesfuscostratus*	Unknown	DK 16_251	OL436970	–
*Leptosporomycesgalzinii*	Sweden	GB 0107211	LR694202	LR694180
*Leptosporomycesgalzinii*	USA	UC 2023126	KP814291	–
*Leptosporomyceslinzhiensis*	China	CLZhao 31174	**PP399152**	**PP862922**
*Leptosporomyceslinzhiensis*	China	CLZhao 31183	**PP399153**	**PP862918**
*Leptosporomyceslinzhiensis*	China	CLZhao 31187	**PP399154**	–
*Leptosporomyceslinzhiensis*	China	CLZhao 31190	**PP399155**	–
*Leptosporomycesraunkiaeri*	USA	UC 2023053	KP814293	–
*Leptosporomycesraunkiaeri*	USA	CFMR: HHB-7628	GU187528	GU187588
*Leptosporomycesseptentrionalis*	USA	UC 2023047	KP814348	–
*Leptosporomycesseptentrionalis*	Sweden	GB 0090937	LR694203	LR694181
*Leptosporomycesseptentrionalis*	Norway	JS 16122	GU187497	–
*Lobuliciumoccultum*	Sweden	KHL13496b	MT340827	–
*Mythicomycescorneipes*	Unknown	AFTOL-972	DQ404393	AY745707
*Phaeoclavulinaflaccida*	Italy	AMB n. 17671	MK796107	MK796156
*Phlebiellachristiansenii*	Finland	KHL 11689	EU118659	–
*Phlebiellavaga*	Sweden	KHL 11065	EU118660	EU118661
*Pilodermafallax*	Finland	CFMR: S-12	GU187535	–
*Plicaturopsiscrispa*	China	LWZ 20201017-11	ON897938	ON885398
*Plicaturopsiscrispa*	Brazil	URM 85888	NR_153926	NG_060427
*Ramariaabietina*	USA	u066	KY510818	–
*Ramariaacrisiccescens*	USA	OSC 112057	KY354738	KY354711
*Ramariaadmiratia*	USA	TENN: 69114	NR_137862	NG_059504
*Ramariaamyloidea*	USA	OSC 69891	EU837196	KP637036
Ramariaapiculatavar.brunnea	USA	CBS:149.74	MH860840	MH872577
*Ramariaaraiospora*	Germany	OSC 108707	EU846298	–
*Ramariaaurantiisiccescens*	USA	OSC 104868	EU837197	–
*Ramariaaurea*	Italy	AMB 18352	MN637783	MN637796
*Ramariabotrytis*	Italy	AMB n. 18201	NR_189799	NG_241889
*Ramariabotrytis*	Argentina	GM 19044	OP177707	OP177871
*Ramariabotrytis*	USA	snf213	AF377055	–
Ramariabotrytisf.musicolor	Italy	ZT Myc 57160	KY626144	–
Ramariabotrytisvar.aurantiiramosa	USA	OSC 140667	JX310410	–
Ramariabotrytisvar.aurantiiramosa	USA	WTU-F-043053	KX574471	–
*Ramariacelerivirescens*	USA	OSC 140471	JX310392	JX269125
*Ramariaclaviramulata*	USA	WTU-F-043055	KX574472	KX671009
*Ramariaconjunctipes*	USA	OSC: 110613	KC346861	–
*Ramariacoulterae*	USA	OSC 69929	EU669320	EU669320
*Ramariadendrophora*	Argentina	GM 20020	OP177716	OP177880
*Ramariadendrophora*	Argentina	GM 19094	OP177715	OP177879
*Ramariafennica*	Italy	AMB n. 17522	MK682678	–
*Ramariaflavescens*	Italy	AMB 17404	KY354743	–
*Ramariaflavescens*	Italy	AMB 17404	MK493036	–
*Ramariaflava*	Italy	AMB 17393	MK493035	–
*Ramariaflavinedulis*	Argentina	GM 19056	OP177717	OP177881
*Ramariaflavinedulis*	Argentina	GM 19035	OP177720	OP177884
Ramariaflavobrunnescensvar.aromatica	USA	AGK 059	JQ408240	–
*Ramariafoetida*	USA	AGK 058	JQ408239	JQ408239
*Ramariaformosa*	USA	OSC1064203	EU525994	–
*Ramariafumosiavellanea*	USA	WTU-F-063048	MK169345	–
*Ramariagelatiniaurantia*	USA	OSC 65737	KP658144	–
*Ramariainedulis*	Chile	12648	OP177723	OP177887
*Ramariainedulis*	Argentina	GM 19047	OP177722	OP177886
*Ramarialargentii*	USA	OSC 67012	KP658130	KP637058
*Ramarialuteovernalis*	Italy	MCVE 28637	NR_155720	KT357477
*Ramariamaculatipes*	USA	OSC 112051	KY354749	KY354721
*Ramariamagnipes*	USA	WTU-F-063057	MK169351	MK493050
*Ramariamyceliosa*	USA	AGK 035	JQ408230	–
*Ramariaobtusissima*	USA	TFB 14473	KJ655554	KJ655575
*Ramariapatagonica*	Argentina	403	OP177710	OP177874
*Ramariapatagonica*	Argentina	GM 19106	OP177713	OP177877
*Ramariapseudoflava*	Italy	AMB 17392	MK493046	–
*Ramariarasilisporoides*	Pakistan	MH-2013	MG760613	–
*Ramariarasilisporoides*	USA	WTU-F-043029	MK169346	–
*Ramariarubella*	USA	OSC 115946	EU669317	EU669343
Ramariarubellaf.rubella	USA	AGK 049	JQ408236	–
*Ramariarubribrunnescens*	USA	OSC 119676	EU652352	EU652387
*Ramariarubribrunnescens*	USA	OSC 66051	KY354750	KY354722
Ramariasandaracinavar.sandaracina	Canada	UBC F28386	KP454028	–
*Ramaria* sp.	India	KD-14-006	KT824242	–
*Ramariastricta*	Germany	CBS 165.48	MH856299	–
Ramariastrictavar.concolor	USA	AGK 011	JQ408221	–
*Ramariastuntzii*	USA	OSC 73315	KP658122	KP637048
*Ramariasubbotrytis*	Spain	MA-Fungi 48088	AJ408361	–
*Ramariasubtilis*	Spain	MA-Fungi 48055	AF442098	–
*Ramariasuecica*	USA	OSC 115933	KP658148	KP637079
*Ramariatestaceoflava*	USA	OSC 107885	KP658128	AY586708
*Ramariaverlotensis*	USA	WTU-F-063047	KX574480	KX671016
*Ramariaxizangensis*	China	CLZhao 31169	**PP399156**	**PP862919**
*Ramariaxizangensis*	China	CLZhao 31180	**PP399157**	**PP862920**
*Ramariaxizangensis*	China	CLZhao 31204	**PP399158**	**PP862921**
*Ramariaformosa*	Italy	AMB 18529	MT055910	MT053203
*Ramariciumpolyporoideum*	USA	TENN: 065654	MF992160	MF992160
*Stereopsisvitellina*	Sweden	F 703241	LR694211	LR694189
*Turbinellusfloccosus*	USA	MO 285170	MN319564	MN319563
*Unilacrymaunispora*	Sweden	UPS F 941268	MN595672	MN595672
*Unilacrymaunispora*	Sweden	UPS F 941277	MN595665	MN593500
*Xenasmatellaardosiaca*	Costa Rica	KHL 12928	EU118658	–
*Xenasmatellaardosiaca*	USA	CBS 126045	MH864060	MH875515

Sequences generated for this study were aligned, with additional sequences downloaded from GenBank. Sequences were aligned using MAFFT v.7 (https://mafft.cbrc.jp/alignment/server/), adjusting the direction of nucleotide sequences according to the first sequence (accurate enough for most cases), and selecting the G-INS-i iterative refinement method (Katoh et al. 2019). Alignments were manually adjusted to maximize alignment and minimize gaps with BioEdit v.7.0.9 (Hall 1999). A dataset of concatenated ITS and LSU sequences was used to determine the phylogenetic position of the new species. Maximum likelihood (ML) analysis was performed using the CIPRES Science Gateway (Miller et al. 2010) based on the dataset using the RA × ML-HPC BlackBox tool, with setting RA × ML halt bootstrapping automatically and 0.25 for maximum hours and obtaining the best tree using ML search. Other parameters in ML analysis used default settings, and statistical support values were obtained using nonparametric bootstrapping with 1,000 replicates. Bayesian inference (BI) analysis based on the dataset was performed using MrBayes v.3.2.6 (Ronquist and Huelsenbeck 2012). The best substitution model for the dataset was selected by ModelFinder (Kalyaanamoorthy et al. 2017) using a Bayesian information criterion, and the model was used for Bayesian analysis. Four Markov chains were run from random starting trees. Trees were sampled every 1,000^th^ generation. The first 25% of sampled trees were discarded as burn-in, whereas other trees were used to construct a 50% majority consensus tree and for calculating Bayesian posterior probabilities (BPPs). The aligned sequences were deposited in TreeBase (https://www.treebase.org/treebase-web/home.html; submission ID 31437).

Branches of the consensus tree that received bootstrap support for ML were greater than or equal to 75%, Bayesian posterior probabilities more than 0.9, respectively.

## ﻿Result

### ﻿The Phylogeny of *Calocera*

**Figure 1.**
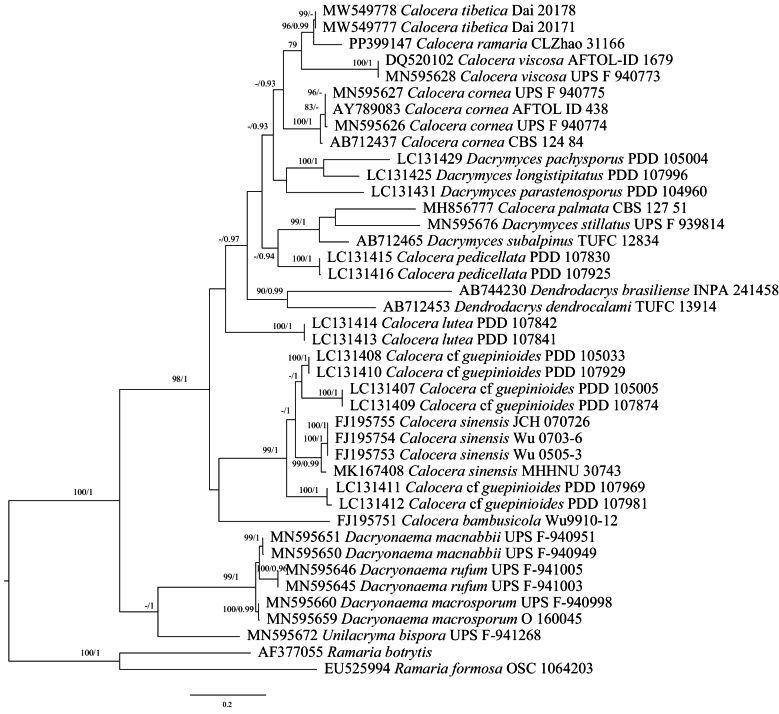
Phylogeny of species in *Calocera* generated by maximum likelihood based on ITS+LSU sequence data. Branches are labeled with maximum likelihood bootstrap ≥ 75% and Bayesian posterior probabilities ≥ 0.90, respectively. New species are in bold.

BI analysis yielded a similar topology to MP and ML analysis. Only the MP tree is provided here (Fig. 1). Branches that received bootstrap support for ML (ML-BS), and BI (BPP) greater than or equal to 75% (MP-BS and ML-BS) and 0.90 (BPP) were considered as significantly supported, respectively. The ITS and LSU dataset contains sequences from 26 fungal specimens representing twelve *Calocera* taxa. The average SD of split frequencies in BI analyses is 0.005504 (BI). The phylogenetic tree (Fig. 1) reveals the new species has close relationship with *C.tibetica*, sister to *C.viscosa* and *C.cornea*.

### ﻿The Phylogeny of *Ceraceomyces*

**Figure 2.**
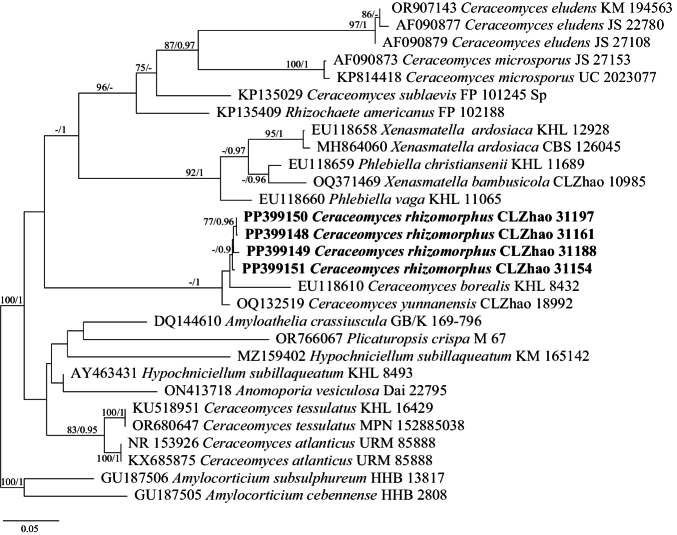
Phylogeny of species in *Ceraceomyces* generated by maximum likelihood based on ITS+LSU sequence data. Branches are labeled with maximum likelihood bootstrap ≥ 75% and Bayesian posterior probabilities ≥ 0.90, respectively. New species are in bold.

The dataset included ITS and LSU from 29 samples representing 22 taxa. The best model for the concatenated ITS+LSU dataset estimated and applied for BI analysis was “GTR+I+G4”, datatype = DNA, nucmodel = 4by4, lset nst = 6, rates = invgamma; state frequencies had a Dirichlet prior (1,1,1,1), and the distribution was approximated using four categories. BI analysis yielded a similar topology to ML analysis, with an average standard deviation of split frequencies of 0.006593. The ML tree was provided (Fig. 2). Branches that received bootstrap support for ML and BI ≥ 70%, and 0.75 were considered significantly supported, respectively.

The analysis reveals four clades (Fig. 2), in which three European species *C.eludens*, *C.microsporus*, *C.sublaevis* clustered together and *Rhizochaeteamericanus* (Nakasone, C.R. Bergman & Burds.) Gresl., Nakasone & Rajchenb. The core clade formed by *C.tessulatus* and *C.atlanticus*, along with *Hypochniciellumsubillaqueatum* (Litsch.) Hjortstam. Four specimens from China formed two lineages, namely *Ceraceomycesrhizomorphus* with *C.yunnanensis*, and were sister to *C.borealis*.

### ﻿The Phylogeny of *Leptosporomyces*

**Figure 3.**
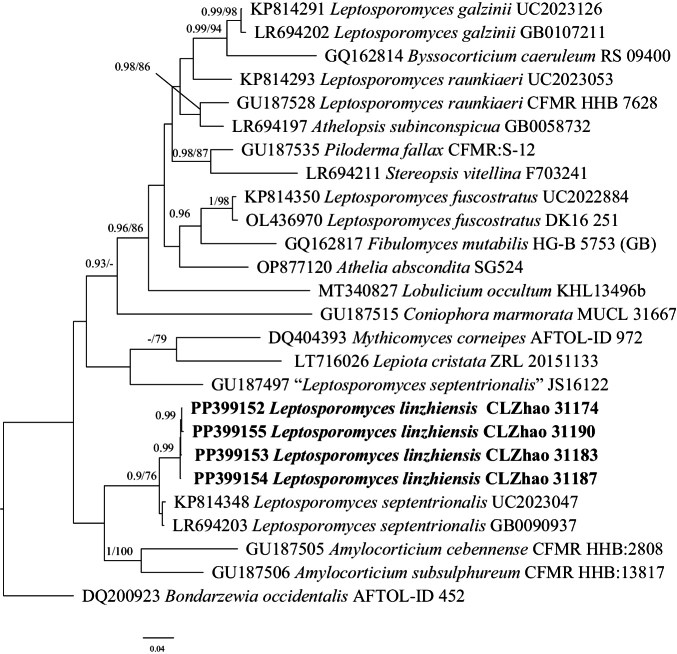
Phylogeny of species in *Leptosporomyces* generated by maximum likelihood based on ITS+LSU sequence data. Branches are labeled with maximum likelihood bootstrap ≥ 75% and Bayesian posterior probabilities ≥ 0.90, respectively. New species are in bold.

BI analysis yielded a similar topology to MP and ML analysis, with an average standard deviation of split frequencies = 0.008841. Only the MP tree is provided here (Fig. 3). Branches that received bootstrap support for ML (ML-BS), and BI (BPP) greater than or equal to 75% (MP-BS and ML-BS) and 0.90 (BPP) were considered as significantly supported, respectively. Four previously accepted species, *L.galzinii*, *L.fuscostratus* (Jülich) Krieglst., *L.raunkiaeri*, and *L.mundus* (H.S. Jacks. & Dearden) Jülich received strong support in three lineages. The new species *L.linzhiensis* had a close relationship with *L.septentrionalis* with full support.

### ﻿The Phylogeny of *Ramaria*

**Figure 4.**
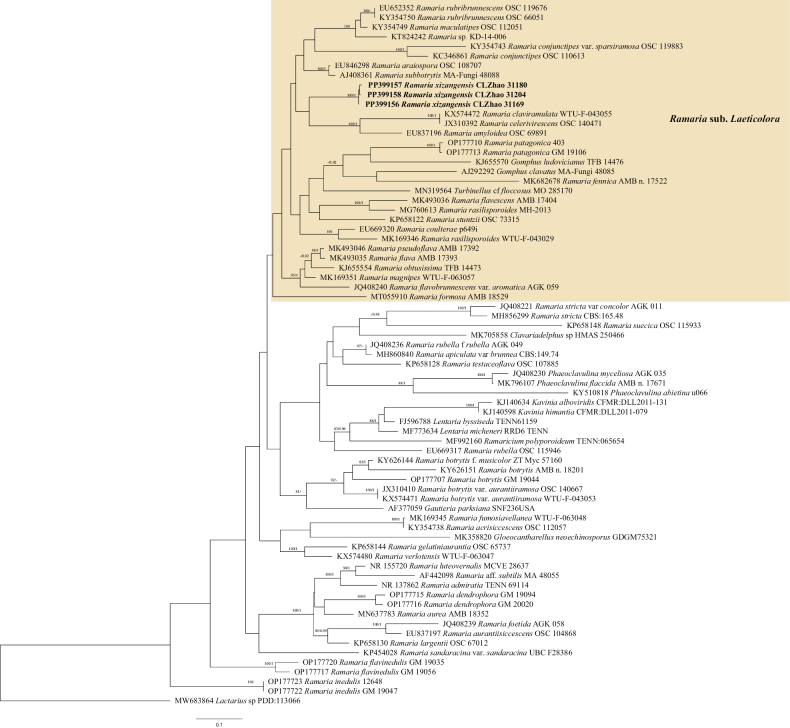
Phylogeny of species in the *Ramaria* generated by maximum likelihood based on ITS+LSU sequence data. Branches are labeled with maximum likelihood bootstrap ≥ 75% and Bayesian posterior probabilities ≥ 0.90, respectively. New species are in bold.

BI analysis yielded a similar topology to MP and ML analysis. Only the MP tree is provided here (Fig. 4). Branches that received bootstrap support for ML (ML-BS), and BI (BPP) greater than or equal to 75% (MP-BS and ML-BS) and 0.90 (BPP) were considered as significantly supported, respectively. Four clades were obtained from our phylogenetic analysis, *Ramaria* sub. *Laeticolora*, *Ramaria* Sub. *Ramaria*, *Ramaria* Sub. *Echinormaria* and *Ramaria* sub. *Lentoramaria*. The species *Ramariaxizangensis* was grouped in *Ramaria* sub. *Laeticolora* along with *R.amyloidea* Marr & D.E. Stuntz, *R.celerivescens* Marr & D.E. Stuntz, and *R.claviramulata* Marr & D.E. Stuntz.

### ﻿Taxonomy

#### ﻿ Calocera ramaria

Taxon classificationFungiDacrymycetalesDacrymycetaceae

C.L. Zhao & H.M. Zhou sp. nov.

EAC05090-4771-50EB-A8DF-C57D797BEE08

852565

Figs 5 , 6

##### Holotype.

China, Xizang, Linzhi, Sejila Mountain National Forest Park, 29°64'N, 94°71'E, elev. 3852 m, gregarious on humus under *Abies*, 2 August 2023, CLZhao 31166 (SWFC).

**Figure 5.**
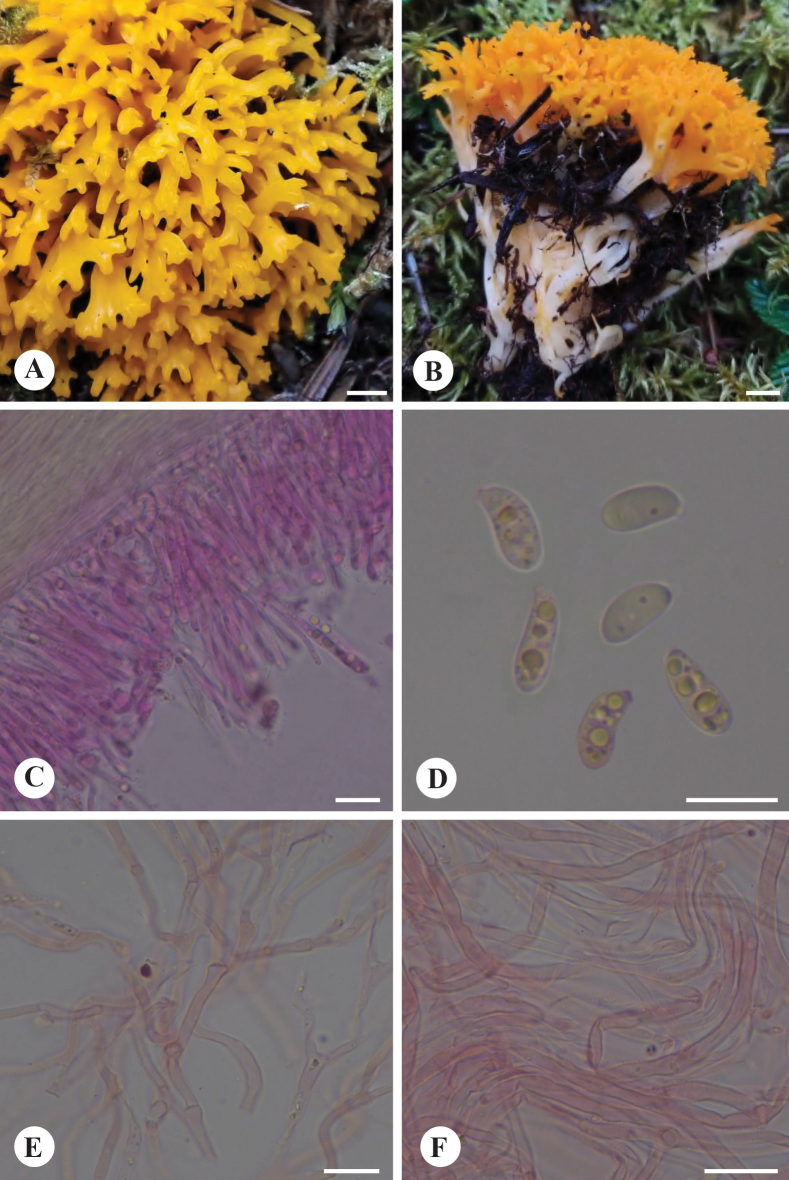
Basidiomata and microscopic structures of *Caloceraramaria* (holotype, CLZhao 31166, holotype) **A, B** basidiomata **C** a section of hymenium **D** basidiospores **E** marginal hyphae **F** internal hyphae. Scale bars: 1 cm (**A, B**); 10 μm (**C–F**).

##### Etymology.

*Ramaria* (Lat.): refers to the ramal basidiomata of the specimens.

**Figure 6.**
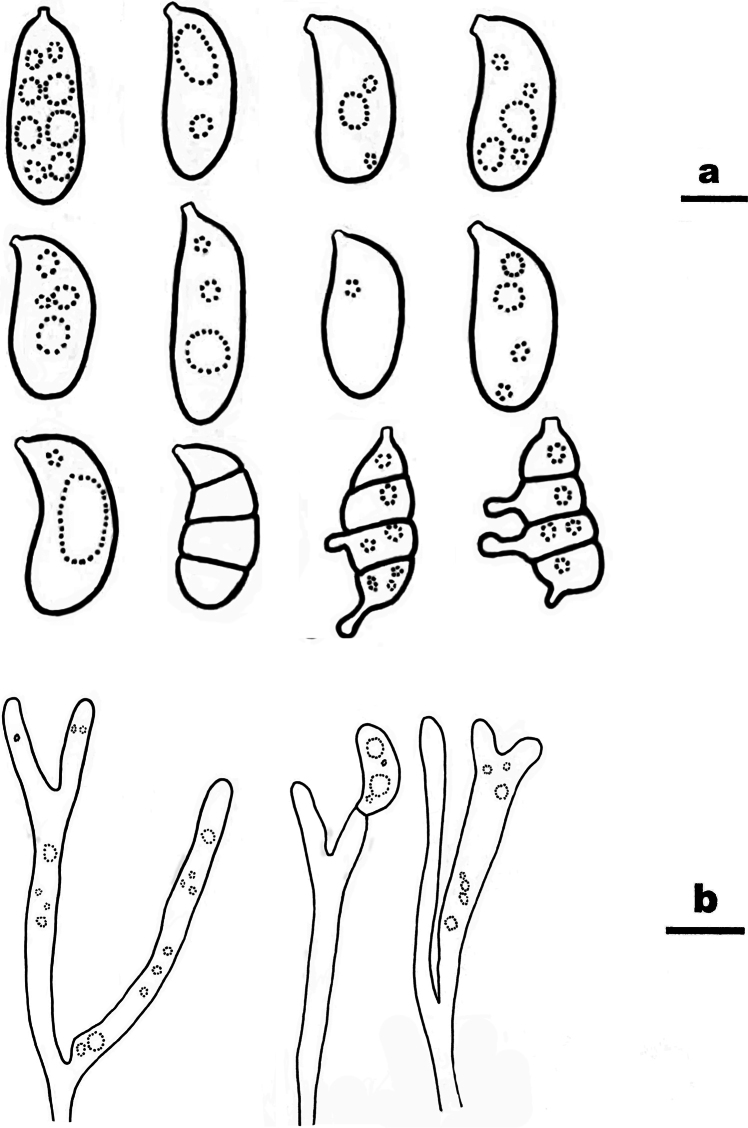
Microscopic structures of *Caloceraramaria* (holotype, CLZhao 31166) **a** basidiospores **b** basidia with basidiospores. Scale bars: 5 μm (**a**); 10 μm (**b**).

##### Diagnosis.

Differed from other species in having ramal basidiomata, septate hyphae, usually 4-septate basidiospores (9.2–11 × 3.9–4.4 μm).

##### Fruiting body.

Basidiomata stipitate, gregarious, bright orange when fresh, orange brown when dry, gelatinous when soaked, corneous when dry, ramal, repeatedly branched, apically blunt, up to 6.2 cm high; stipe 0.7–1 mm in diam, become orange to reddish brown corneous when dry.

##### Internal features.

Marginal hyphae hyaline, smooth, thin-walled, septate, simple or branched, without clamp connections, 4–5.5 μm in diam; internal hyphae hyaline, smooth or scabrous, thin- to slightly thick-walled, interwoven, with nodose-septa, without clamp connections, 2–3 μm in diam; hyphidia hyaline, smooth, thin-walled, with a simple septum at base, occasionally terminally branched; basidia hyaline, thin-walled, subclavate to clavate, without basal clamp connection, 23–31 × 2–4 μm; basidiospores hyaline, smooth, thin-walled, oblong-ellipsoid to navicular, straight or curved, apiculate, usually 4-septate when mature, occasionally 5-septate, (9.1–)9.2–11(–11.6) × (3.5–)3.9–4.4(–4.7) μm, L = 10.18 μm, W = 4.19 μm, Q = 2.43 (n = 30/1).

#### ﻿ Ceraceomyces rhizomorphus

Taxon classificationFungiPolyporalesPhanerochaetaceae

C.L. Zhao & H.M. Zhou sp. nov.

6996A41F-4C27-5145-ACB9-B73FEA82AE09

852584

Figs 7 , 8

##### Holotype.

China, Xizang, Linzhi, Sejilashan National Forest Park, 29°64'N, 94°71'E, elev. 3848 m, on the fallen branch of *Abies*, 2 August 2023, CLZhao 31188 (SWFC).

**Figure 7.**
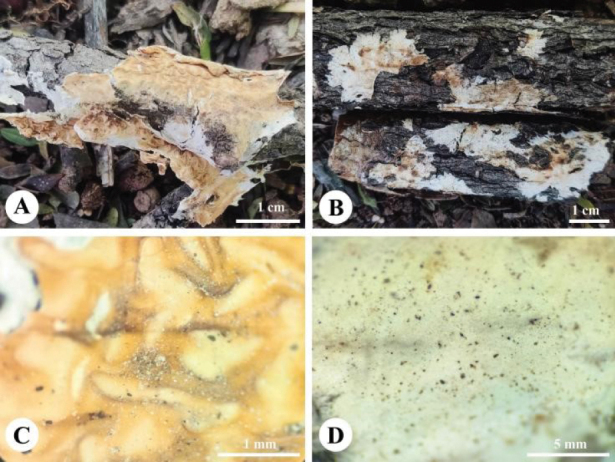
Basidiomata of *Ceraceomycesrhizomorphus***A, C** CLZhao 31188 (holotype) **B, D** CLZhao 31185.

##### Etymology.

*Rhizomorphus* (Lat.): refers to the basidiomata with rhizomorphs.

**Figure 8.**
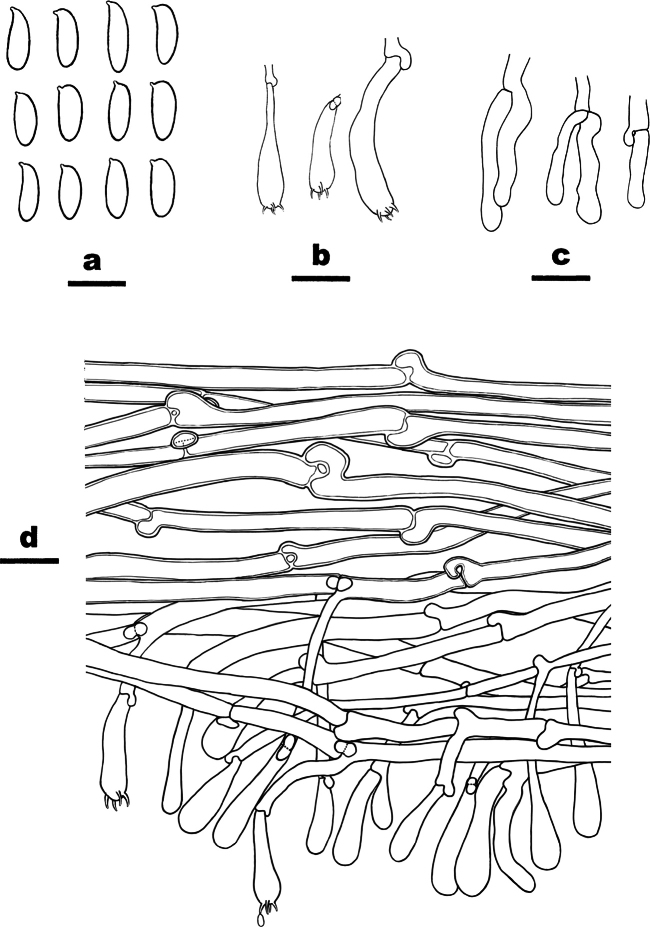
Microscopic structures of *Ceraceomycesrhizomorphus* (holotype, CLZhao 31216) **a** basidiospores **b** basidia **c** basidioles **d** a section of hymenium. Scale bars: 5 μm (**a**); 10 μm (**b–d**).

##### Diagnosis.

Differed from other species in having merulioid, cream to yellowish basidiomata, generative hyphae with clamp connections, cylindrical basidiospores (4.7–6.2 × 1.8–2.3 µm).

##### Fruiting body.

Basidiomata resupinate, adnate, smooth to tuberculate when fresh, merulioid upon drying, without odor or taste when fresh, up to 6 cm long, 2 cm wide, 100–200 µm thick. Hymenial surface merulioid, cream to yellowish when fresh, turn to orange yellow upon drying. Margin sterile, white, with rhizomorphs.

##### Hyphal structure.

Hyphal system monomitic, generative hyphae with clamp connections, colorless, thin- to slightly thick-walled, branched, interwoven, 3.5–7 µm in diameter, IKI–, CB–; tissues turn black in KOH.

##### Hymenium.

Cystidia and cystidioles absent; basidia narrowly clavate to clavate, in a dense palisade, with 4 sterigmata and a basal clamp connection, 16–19 × 3.5–4 µm; basidioles dominant, similar to basidia in shape, but slightly smaller.

##### Spores.

Basidiospores cylindrical, with suprahilar depression, colorless, smooth, thin-walled, IKI–, CB–, (4.2–)4.7–6.2(–6.4) × (1.5–)1.8–2.3(–2.4) µm, L = 5.49 µm, W = 2.05 µm, Q = 2.66–2.68 (n = 60/2).

##### Additional specimens examined

**(*paratypes*).** China. Xizang, Linzhi, Sejila Mountain National Forest Park, 29°64'N, 94°71'E, elev. 3848 m, on the trunk of *Abies*, 2 August 2023, CLZhao 31153 (SWFC); CLZhao 31154 (SWFC); CLZhao 31161 (SWFC); CLZhao 31202 (SWFC); on the fallen branch of *Abies*, 2 August 2023, CLZhao 31184 (SWFC); CLZhao 31185 (SWFC); CLZhao 31197 (SWFC).

#### ﻿ Leptosporomyces linzhiensis

Taxon classificationFungiAthelialesAtheliaceae

C.L. Zhao & H.M. Zhou sp. nov.

D349945D-8A6F-5306-AE04-AEAC502F1749

852585

Figs 9 , 10

##### Holotype.

China, Xizang, Linzhi, Sjilashan Forest Park, 29°64'N, 94°71'E, elev. 3848 m, on fallen trunk of *Abies*, 2 August 2023, CLZhao 31183 (SWFC).

**Figure 9.**
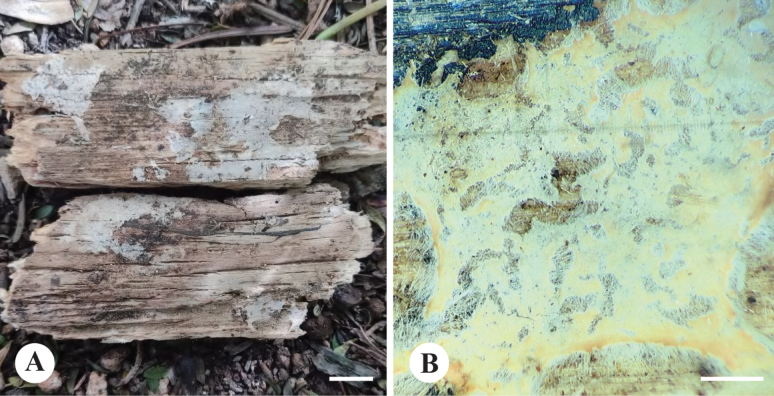
Basidiomata of *Leptosporomyceslinzhiensis* (holotype, CLZhao 31183). Scale bars: 1 cm (**A**); 1 mm (**B**).

##### Etymology.

*Linzhiensis* (Lat.): refers to the locality (Xizang) of the type specimens.

**Figure 10.**
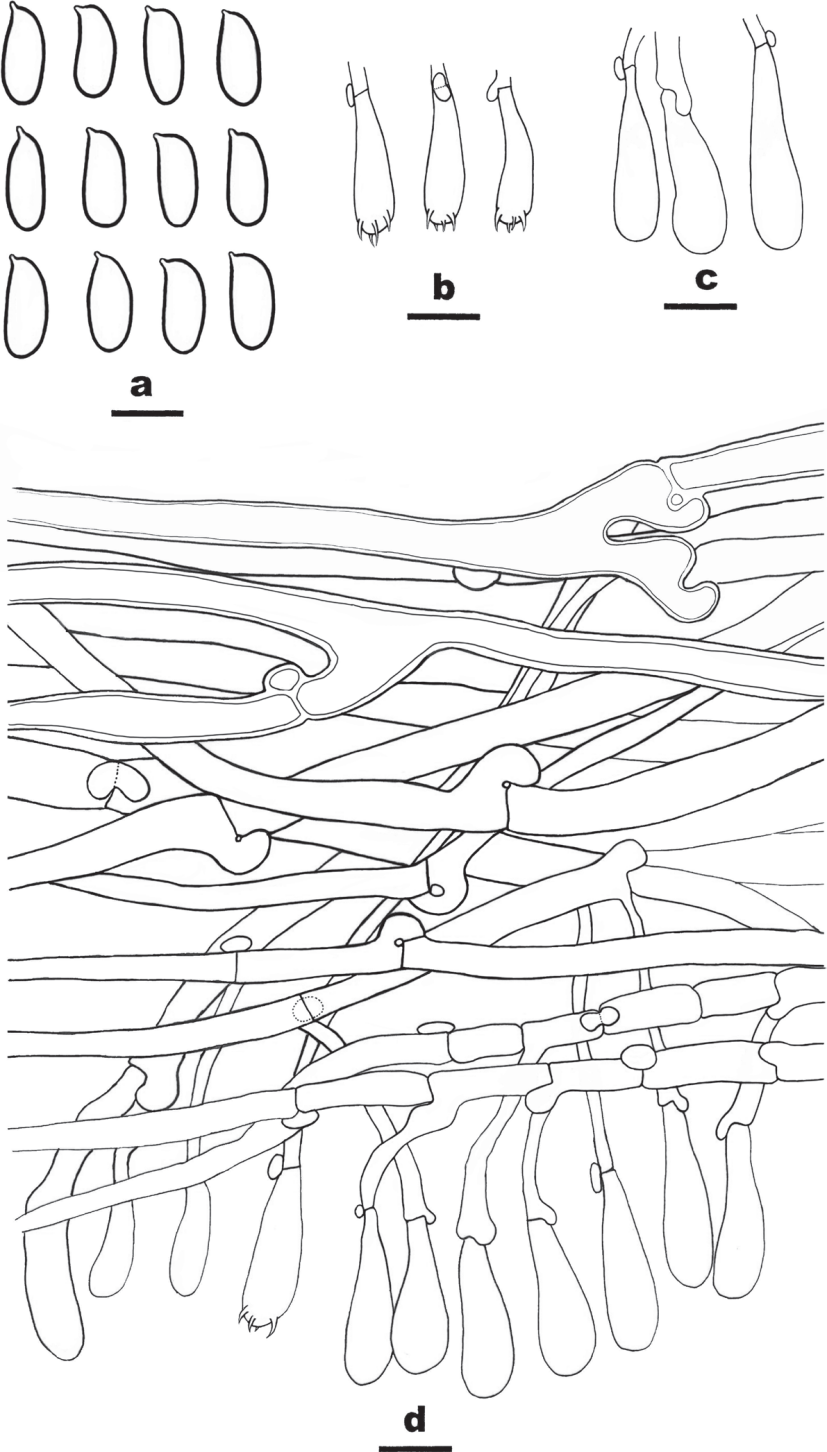
Microscopic structures of *Leptosporomyceslinzhiensis* (holotype, CLZhao 31183) **a** basidiospores **b** basidia **c** basidioles **d** A section of hymenium. Scale bars: 5 μm (**a**); 10 μm (**b–d**).

##### Diagnosis.

Differed from other species in having white basidiomata, monomitic hyphal system, cylindrical to oblong ellipsoid basidiospores (3.8–4. × 1.7–2 µm).

##### Fruiting body.

Basidiomata resupinate, athelioid, membranous upon drying, without odor or taste when fresh, up to 10 cm long, 4 cm wide, 200 µm thick. Hymenial surface smooth to cracked, white with pink tint when fresh, turning to yellowish cream upon drying. Margin sterile, white, fimbriate.

##### Hyphal structure.

Hyphal system monomitic, generative hyphae with clamp connections, colorless, thin- to slightly thick-walled, branched, interwoven, 2–5 µm in diameter, IKI–, CB–; tissues turn black in KOH.

##### Hymenium.

Hyphal system monomitic, generative hyphae with clamp connections, colorless, thin-walled, branched, interwoven, 2–3.5 µm in diameter, IKI–, CB–. Basidia clavate, with 4 sterigmata and a basal clamp connection, 11.5–13.5 × 3.2–3.8 µm.

##### Spores.

Basidiospores cylindrical to oblong ellipsoid, colorless, smooth, thin-walled, IKI–, CB–, (3.5–)3.8–4.3(–4.7) × (1.7–)1.7–2(–2.3) µm, L = 4.02 µm, W = 1.88 µm, Q = 1.95–2.18 (n = 90/3).

##### Additional specimens examined

**(*paratypes*).** China, Xizang, Linzhi, Sjilashan Forest Park, 22°57'N, 103°42'E, elev. 2100 m, on fallen trunk of *Abies*, 2 August 2023, CLZhao 31174 (SWFC); on fallen trunk of *Abies*, 2 August 2023, CLZhao 31187 (SWFC); on fallen trunk of *Abies*, 2 August 2023, CLZhao 31190 (SWFC).

#### ﻿ Ramaria xizangensis

Taxon classificationFungiGomphalesGomphaceae

C.L. Zhao & H.M. Zhou sp. nov.

9939FB18-1A60-5780-B821-1FC76B01C776

852586

Figs 11 , 12

##### Holotype.

China, Xizang, Linzhi, Sejila Mountain National Forest Park, 29°64'N, 94°71'E, elev. 3850 m, gregarious on the humus under *Abies*, 2 August 2023, CLZhao 31169 (SWFC).

**Figure 11.**
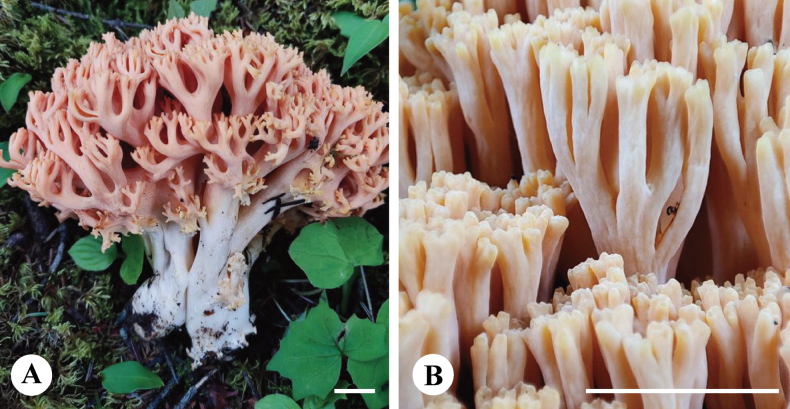
Basidiomata of *Ramariaxizangensis* (holotype, CLZhao 31169). Scale bars: 1 cm (**A, B**).

##### Etymology.

*Xizangensis* (Lat.): refers to the locality (Xizang) of the type specimens.

**Figure 12.**
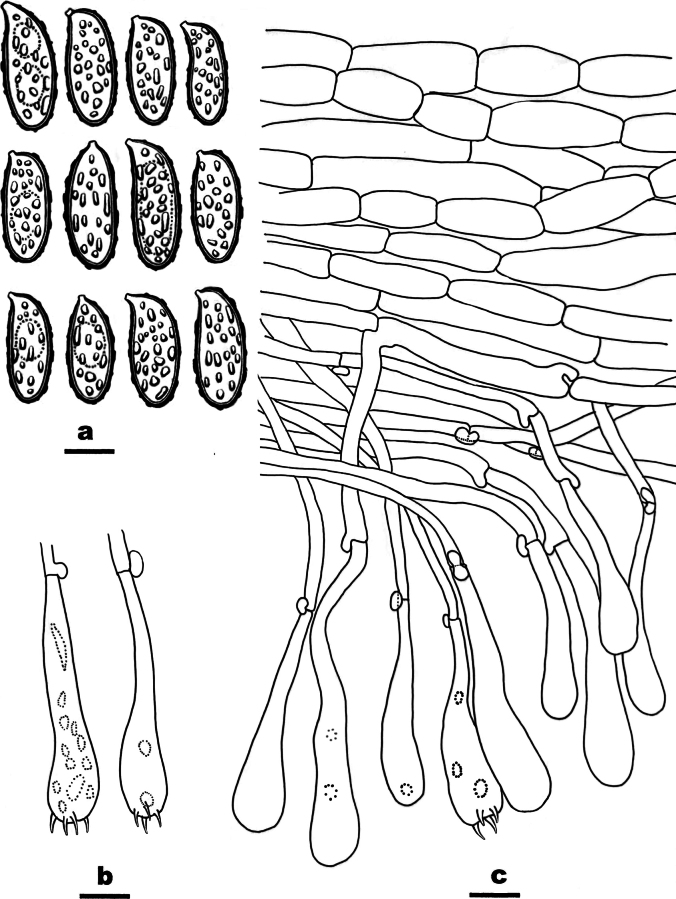
Microscopic structures of *Ramariaxizangensis* (CLZhao 31169, holotype) **a** basidiospores **b** basidia **c** a section of hymenium. Scale bars: 5 μm (**a**); 10 μm (**b, c**).

##### Diagnosis.

Differed from other species in having flesh pink basidiomata, monomitic hyphal system, generative hyphae with clamp connections, ellipsoid to cylindrical, densely warted basidiospores (9.7–11.8 × 3.9–4.9 µm).

##### Fruiting body.

Basidiomata solitary to gregarious, with 8 cm high × 6 cm wide at the widest point, repeat branched dichotomously in 4–5 ranks, flesh pink when fresh, become clay buff with dry; apices obtuse, orange yellow when fresh, becoming fuscous when dry. Stipe ≥ 3 cm high, compound to fasciculate in groups of 5, emerging from a common base, concolorous with the branches.

##### Hyphal structure.

Hyphal system monomitic, generative hyphae with clamp connections, branched, walls smooth and hyaline; basal stem with tramal hyphae 4–7 μm wide and inflated ones up to 10 μm, occasionally branched, thin-walled, parallel arranged, hyaline; tramal hyphae of branches 3–4 μm wide.

##### Hymenium.

Hymenium all along the basidiomata. Basidia clavate, in a dense palisade, with 4 sterigmata and a basal clamp connection. Basidioles elongated clavate, smooth, hyaline, contents homogeneous, 23.5–34 × 6–7 μm.

##### Spores.

Basidiospores ellipsoid to cylindrical, densely warted, with 1–2 several guttulae, IKI–, CB–, 9.7–11.8(–12.5) × (3.8–)3.9–4.9(–5.1) µm, L = 10.69 µm, W = 4.29 µm, Q = 2.49 (n = 30/1).

##### Additional specimens examined

**(*paratypes*).** China, Xizang, Linzhi, Sejila Mountain National Forest Park, 29°67'N, 94°74'E, elev. 3850 m, gregarious on the humus under *Abies*, 2 August 2023, CLZhao 31180 (SWFC); on ground in forest of *Abies*, 2 August 2023, CLZhao 31204 (SWFC).

## ﻿Discussion

Wood decay fungi encompasses the vast group of aphyllophoroid fungi with corticioid, prioid or jelly form of basidiomata (Herter 1910). This classification has historically been used to define the different families of Basidiomycetes. However, molecular studies have revealed that many of these fungi are distributed across various orders within the Basidiomycetes, including the likes of Amylocorticiales, Atheliales, Dacrymycetales, and Gomphales (Kirk et al. 2018; Wei et al. 2022). As a result, further research is needed to elucidate the relationships and morphological variability of these taxa through phylogenetic analysis.

The Xizang Autonomous Region, situated in the southwest of China, is renowned as one of the most bio-diverse regions in the country. This is attributed to its complex topography and diverse ecosystems, making it a focal point for fungal biodiversity in China. Recently, studies focusing on fungal diversity and the ecology of Basidiomycota in Xizang were carried out (Ke 2016; Pubu et al. 2016; Wang et al. 2023). According to the study (Pubu et al. 2016), 1733 species were collected in Xizang. The fungal research indicated that Sejila Mountain National Forest Park is predominantly composed of spruce and fir trees, which provide an ideal habitat for a rich diversity of macrofungi species to flourish (Zhao and Li 1987). In our study, four species were found from Xizang, *Caloceraramaria*, *Ceraceomycesrhizomorphus*, *Leptosporomyceslinzhiensis*, and *Ramariaxizangensis*.

*Calocera* is characterized by its yellow, gelatinous basidiomata, resembling *Dacrymyces*. However, *Dacrymyces* displays a broader range of basidiomata forms, including pulvinate, discoid, turbinate, spathulate, flabellate, and cylindrical shapes (Shirouzu et al. 2009; Fan et al. 2021), whereas *Calocera* exhibits branched, dendroid basidiomata. Our results have further confirmed that our newly discovered species features ramal basidiomata and clusters phylogenetically with *Calocera* species, placing it within the genus *Calocera*. In Xizang, two species have been identified, *C.ramaria* and *C.tibetica*, but the latter has wider basidiospores (5–6 µm vs. 3.9–4.4 µm, Fan et al. 2021). In our phylogenies, *C.viscosa* and *C.cornea* were related to *C.ramaria* (Fig. 1); however, *C.viscosa* has 1-septate mature basidiospores, and *C.cornea* differs from *C.ramaria* by its distinctly larger basidiospores (7–10 × 3–4.5 μm vs. 9.2–11 × 3.9–4.4 μm) with one septum (McNabb 1965; Shirouzu et al. 2009).

Previous research has highlighted the polyphyly of *Ceraceomyces* (Chikowski 2016; Yuan et al. 2023), and seven species are retained in *Ceraceomyces*. However, it is worth noting that authentic specimens and DNA data are lacking for *Ceraceomyces* species. Phylogenetically, *C.rhizomorphus* formed a sister group with *C.yunnanensis* and *C.borealis*, but *C.yunnanensis* has smaller basidiospores (3–4 × 1–1.5 µm vs. 4.7–6.2 × 1.8–2.3 µm, Yuan et al. 2023) and *C.borealis* has larger basidiospores (6–8 × 1.8–2 µm vs. 4.7–6.2 × 1.8–2.3 µm, Bernicchia and Gorjón 2010).

*Ceraceomycesrhizomorphus* and *C.tessulatus* had similar yellowish basidiomata with rhizomorphs when fresh, while *C.tessulatus* has ellipsoid and larger basidiospores (6–8 × 3.5–4.5 µm vs. 4.7–6.2 × 1.8–2.3 µm, Bernicchia and Gorjón 2010). Three known species, *C.bizonatus*, *C.reidii*, and *C.simulans* also distributed in Asia. However, *C.bizonatus* has shorter basidiospores (2.5–3.3 µm vs. 4.7–6.2 µm, Bernicchia and Gorjón 2010); *C.reidii* has larger basidiospores (11.5–15 × 4.5–6 µm vs. 4.7–6.2 × 1.8–2.3 µm, Bernicchia and Gorjón 2010); *C.simulans* has longer basidiospores (6–7 µm vs. 4.7–6.2 µm, Bernicchia and Gorjón 2010).

*Leptosporomyceslinzhiensis* is similar to *L.thindii* in having white basidiomata and being distributed in Asia, but the latter has wider basidiospores (Prasher 2015). *Leptosporomyceslinzhiensis* sisters to *L.septentrionalis* by its white basidiomata, and cylindrical basidiospores, but the latter has slightly shorter basidiospores (3–4 μm vs. 3.8–4.3 μm), and 2–4 basidia (Prasher 2015). *Leptosporomyceslinzhiensis* is easily confused with *L.roseus* in, but the latter has shorter basidiospores (2–2.5 μm vs. 3.8–4.3 μm, Prasher 2015). *Leptosporomycesfuscostratus* has a broad distributional range in the northern hemisphere, but it has wider basidiospores (2–2.8 μm vs. 1.7–2 μm, Yurchenko and Wołkowycki 2022).

In our phylogeny, *Ramaria* is paraphyletic, which included four clades, *R.* sub. *Laeticolora* and *R.* sub. *Lentoramaria*, *R.* sub. *Ramaria* and R. sub. *Echinormaria*. *Ramariaxizangensis* was clustered in *Ramaria* sub. *Laeticolora* with *Ramariaamyloidea*, *R.celerivirescens* and *R.claviramulata*. However, *R.celerivirescens* has slightly wider basidiospores (4–6 µm vs. 3.9–4.9 µm, Marr and Stuntz 1973). *R.claviramulata* has cream to brownish white basidiomata. *Ramariaxizangensis* is similar to *R.indoyunnaniana* in having pink basidiomata and being distributed in Yunnan, but the latter has shorter basidiospores (7.2–8.3 µm vs. 9.7–11.8 µm, Petersen and Zang 1986).

According to our field inventory, the four Chinese new species were found in alpine zone near the Sejila Mountain, and the coniferous forest dominant by *Abies* at high altitude with cold and humid environments. Previously, numerous new species have been found in Southwest China (Dai 2022; Zhao et al. 2023), and the present paper confirms the fungal diversity is very rich in the montane forests of Southeast Xizang.

## Supplementary Material

XML Treatment for Calocera ramaria

XML Treatment for Ceraceomyces rhizomorphus

XML Treatment for Leptosporomyces linzhiensis

XML Treatment for Ramaria xizangensis

## ﻿Additional information

### Author ORCIDs

Hong-Min Zhou https://orcid.org/0000-0002-0724-5815

Xun-Chi Zhang https://orcid.org/0000-0003-3887-0979

Fang Wu https://orcid.org/0000-0002-1455-6486

Chang-Lin Zhao https://orcid.org/0000-0002-8668-1075

### Data availability

All of the data that support the findings of this study are available in the main text.
